# Endoscope disinfectant-induced colonic pseudolipomatosis: case series of a rare condition

**DOI:** 10.2144/fsoa-2023-0210

**Published:** 2024-06-12

**Authors:** Charfeddine Baccouche, Myriam Ayari, Imen Abdelaali, Amen Dhaoui, Taieb Jomni, Mohamed Hedi Douggui

**Affiliations:** 1 Gastroenterology Department, Internal Security Forces Hospital La Marsa, Tunis, 2070, Tunisia; 2 Pathology Department, Internal Security Forces Hospital La Marsa, Tunis, 2070, Tunisia; 3 University of Tunis El Manar, Tunis, 1068, Tunisia

**Keywords:** colon, disinfectant, endoscopy, mucosa, pseudolipomatosis

## Abstract

**Aim:** Colonic mucosal pseudolipomatosis is a rare and benign endoscopic finding with distinct macroscopic and histological characteristics. **Case series:** We observed a form of unprecedented colitis in eight patients in a 3-month period. Operators have found, during colonoscopy, flat or slightly raised whitish-yellow plaques, in the colonic mucosa of all patients. Histological examination concluded to pseudolipomatosis. After investigation, the disinfectant machine was found to have technical malfunctioning of the rinse cycle of the endoscope during this period. No other cases were observed after the machine was fixed. **Conclusion:** Pseudolipomatosis is more an endoscopically induced lesion than a true pathological condition. A careful check of the disinfection process should be carried out when such lesions are detected.

## Plain language summary

Pseudolipomatosis is a rare and harmless condition that can occur in various parts of the digestive system. It looks like flat or slightly raised whitish or yellow patches mixed with normal gut tissue. Under a microscope, it appears as empty spaces in the tissue layer. We found several cases during colonoscopy over a 3-month period, likely caused by the disinfectant used on the endoscope. Finding these lesions should prompt careful inspection of the disinfection procedure.

## Abstract (plain language summary)

Colonic mucosal pseudolipomatosis is a rare and benign endoscopic finding.

At histology, pseudolipomatosis appears as optically empty of variable size spaces in the lamina propria.

Pathogenesis is controversial, including mechanical or chemical injury such as disinfectants.

Induced pseudolipomatosis during endoscopy should incite to a careful check of the disinfection process.

Colonic mucosal pseudolipomatosis is a rare and benign condition described for the first time by Snover *et al.* [1]. Histologically, pseudolipomatosis appears as an optically empty of variable size spaces in the lamina propria [2,3]. The pathogenesis of this condition is controversial. Several mechanisms have been postulated, including chemical injury by disinfectants [4,5]. Other authors have suggested that intramucosal entrapment of air by mechanical injury during insufflation, biopsy or invasive procedures contributes to the pathogenesis of these lesions [4,6,7]. Here in, we report and discuss a case series of patients presenting with colonic pseudolipomatosis during routine colonoscopy.

## Case series presentation

Approximately 1000 colonoscopies are performed per year for inpatients and outpatients in our endoscopy unit. During a 3-month period in late 2021 (October–December), five different endoscopists observed a form of colitis displaying characteristic snow-white patterns in eight patients with a mean age of 58.63 years and a sex-ratio F/M of 1.6 with normal physical examination. Lesions were observed with the different used endoscopes. Table 1 summarizes indications of the colonoscopy, clinical and endoscopic characteristics of the patients.

**Table 1.** Clinical and endoscopic characteristics of patients.

Patient	Age (years)	Sex	Medical history	Indication of colonoscopy	Site of the lesion	Extent of the lesion	Previous colonoscopy
1	53	M	–	Colonic diverticulitis	Right colon	0.5–1 cm	No
2	79	F	Hypertension	Constipation	Right colon (Caecum)	4–5 cm	No
3	68	F	Lupus Hypertension Diabetes	Iron deficiency	Left colon	8–10 cm	No
4	34	F	Basedow's disease	Iron deficiency anemia	Right colon	4–5 cm	No
5	62	F	Coronary artery disease Diabetes	Iron deficiency anemia	Right colonic flexure	5–6 cm	No
6	66	M	Atrial fibrillation Hypertension	Constipation Rectal bleeding	Transverse colon	10–12 cm	6 months earlier
7	50	M	–	Rectal bleeding	Transverse colon	5–6 cm	24 h earlier
8	57	F	–	Constipation	Left colonic flexure	8–10 cm	No

**Figure 1.**
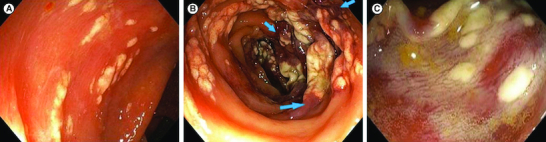
Endoscopic aspects. **(A)** Slightly elevated whitish–yellow plaques. **(B)** Confluent plaques creating a pseudomembranous aspect, sometimes surrounded by erythematous colonic mucosa (blue arrows). **(C)** Formation of plaques rapidly after the appearance of whitish foamy fluid.

At endoscopic examination, flat or slightly elevated lesions, measuring from few millimeters to several centimeters (mean diameter: 68.12 mm), were found in the eight patients with whitish–yellow plaque aspect (Figure 1A). Plaques were in four cases confluent, creating a pseudomembranous appearance, sometimes surrounded by erythematous colonic mucosa (Figure 1B). Lesions were found as the endoscope was being advanced through the colon in two patients. In three patients, lesions were seen when the endoscope was removed.

In the other three patients, a whitish foamy fluid just appeared under our observation rapidly followed by the formation of whitish plaques (Figure 1C). This phenomenon occurred at the moment when water was irrigated using an automated pump. Polypectomy was performed in two patients but whitish plaque lesions were noticed before any intervention was done. Two patients have undergone a previous colonoscopy but neither biopsies nor any other invasive procedures were done in both cases.

**Figure 2.**
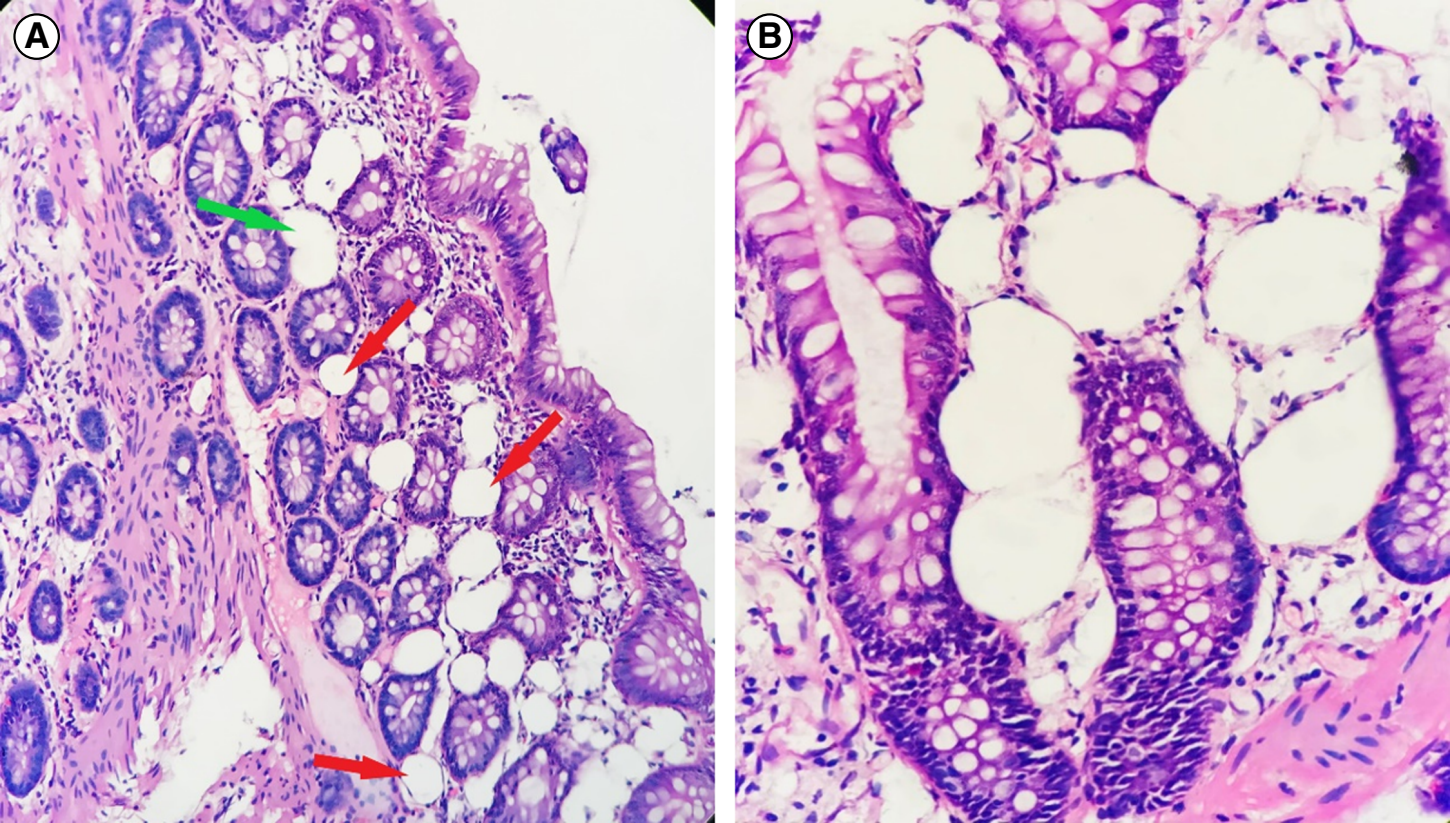
Histological examination. **(A)** Numerous optically empty vacuoles of variable sizes (red arrows) in the lamina propria and the muscularis mucosa, sometimes confluent (green arrow), intermingled with inflammatory cells (hematoxylin-eosin 200×). **(B)** The optically empty vacuoles repress and distort the cryptic contours (hematoxylin-eosin 400×).

At histological examination, biopsy specimens showed the same findings in all cases. Numerous optically empty cavities were observed in the lamina propria and extended to the muscularis mucosa. These clear empty spaces appeared as unilocular vacuoles of variable size (10–50 μm) sometimes confluent, and were environed by a mononuclear inflammatory infiltrate (Figure 2A) suggestive of pseudolipomatosis.

Main differential diagnosis were lipomatosis and lymphatic vessels but the mature adipocyte is a clear spherical cell with well-defined cytoplasmic outlines and a clearly visible peripheral nucleus; as for lymphatic vessels, their endothelial lining is generally easily seen. Moreover, lymphatic vessels and adipocytes are an integral part of the tissue and therefore respect the crypts. This was not the case in our specimens as the optically empty vacuoles push and distort the cryptic contours (Figure 2B).

In view of the histological characteristics and endoscopic features, the diagnosis of pseudolipomatosis was retained in all the patients. None of the patients required medical treatment.

During follow-up, no control colonoscopy was performed, however, none of the patients presented with symptoms nor clinical findings suggesting a pejorative outcome in clinical follow-up. The occurrence of this unprecedented cluster of similar cases led us to investigate a possible cause. The disinfectant we use at our center is composed of a mix of hydrogen peroxide (<5%), acetic acid (<3.5%) and peracetic acid (<0.25%). We noticed that our endoscope washer and disinfector machine had a technical malfunction during the endoscope channels rinse cycles at that period. In fact, a problem with the fluid delivery system was identified resulting in inadequate delivery of rinsing solution to the channels resulting in persisting of residual disinfectant. After the problem was solved, no further cases were reported. None of the patients subsequently presented morbidity or mortality secondary to this form of colitis.

## Discussion

Pseudolipomatosis is a rare and benign condition that may be seen in different parts of the GI tract [3,6,8]. Same cases were also reported in the female genital tract [9]. The frequency of colon pseudolipomatosis ranges from 0.02 to 1.7% in endoscopic series [1,4,10]. No gender predominance was established in adults having this condition [3,5,11] but a slight female predominance was noted in our series. Pseudolipomatosis of the large intestine is usually asymptomatic or can be observed in patients with transient mild symptoms such as rectal bleeding, abdominal pain or bowel movement disorders [4,5,11].

Colon pseudolipomatosis has a very characteristic appearance of flat or slightly elevated whitish or yellow plaques interspersed with normal colonic mucosa, also described as the ‘snow white sign’ [1,3,12,13]. These lesions are often multiple and sometimes confluent [5,13]. However, this typical endoscopic pattern may be absent [14]. This condition, which is unfamiliar to endoscopists due to its rarity, must be distinguished from similar lesions like lipomatosis, pseudomembranous colitis or malakoplakia [2,4,12,15].

Histopathologically, pseudolipomatosis is typically characterized by numerous optically empty vacuolar spaces of variable size in the lamina propria. These vacuoles are usually lined by inflammatory cells with preserved glandular structures, suggesting vascular structures or adipocytes [1,3,16,17]. Intestinal lymphangioma, lipoma or sclerolipomatosis seen in inflammatory bowel disease may mimic histological patterns of pseudolipomatosis [16,18–20]. Immunohistochemistry can be helpful to rule out differential diagnosis as the cells surrounding empty cavities in pseudolipomatosis do not truly express vascular markers (CD31, CD34, D2–40, factor VIII) or adipocytic markers (S-100 protein) [1,5,16]. In our series, immunohistochemistry was deemed unnecessary as the diagnosis was easily made by confronting clinical, endoscopic and histopathologic findings.

Lesions usually show spontaneous regression within 1 to 20 months [5,7,11,12]. However, cases of pneumatosis intestinalis, subcutaneous emphysema and pneumoperitoneum have been reported in association with pseudolipomatosis [13,21]. Overall, conservative management seems to be an acceptable option for colon pseudolipomatosis [22]. This was the case of our patients of which none subsequently suffered morbidity or mortality.

The pathogenesis of pseudolipomatosis is controversial. Several mechanisms have been hypothesized such as penetration of the luminal gas to the bowel wall due to a mucosal injury induced by stretching, abrasive trauma, overinflation, biopsies and other invasive procedures during endoscopy [4,6,7]. The role of microorganisms has also been suggested in the pathogenesis of these lesions. It has been suggested that pseudolipomatosis may be caused by intestinal bacteria releasing gas [11,17]. Iwamuro *et al.* reported a case of a patient presenting with colonic pseudolipomatosis following cytomegalovirus colitis [23]. Cases of gastric pseudolipomatosis have also been described and connected to *Helicobacter pylori* infection [8,24]. More recently, a case of colonic pseudolipomatosis with pneumatosis intestinalis was also reported by Naito *et al.* and was linked to immune checkpoint inhibitor-related colitis [25]. Other authors hypothesized that disinfectants, especially hydrogen peroxide and peracetic acid, have a role in the pathogenesis of this condition [4,5,12]. Colonic pseudolipomatosis was also experimentally induced in pig and murine models after exposure to hydrogen peroxide [26,27]. Cammarota *et al.* reported that pseudolipomatosis developed when the insufflation button was depressed during colonoscopy [5]. The authors suggested that residual hydrogen peroxide in the colonoscope was released after insufflation of air and/or water injection, causing the appearance of mucosal pseudolipomatosis [5,12].

In our case series, it is unlikely that pseudolipomatosis lesions were related to mechanical injuries of the colonic mucosa or were endoscopist-dependent because no invasive procedures preceded the appearance of the lesions and five different endoscopists observed them. Infectious or drug-related causes are also unlikely in our series as history and clinical/biological findings were not suggestive of such mechanisms.

Pseudolipomatosis observed in our series resulted, very probably, from a technical problem in the rinse cycles of the air/water channel and/or the instrument channel as some lesions were observed using both colonoscopes assisted or not by a flashing automated water pump. This led to vaporization of residues of peroxide derivatives during insufflations or water injection resulting in chemical injury of the colonic mucosa. In fact, characteristic lesions appeared under our observation in some patients, when water was irrigated. Colonic pseudolipomatosis lesions were observed only during a brief period and did not happen again after fixing the automatic disinfecting machine. We did not observe any further cases after correction of this technical issue.

No similar cases were seen among patients who were undergoing upper digestive endoscopy even though gastroscopes at our center are bring disinfected using the same machine. This is likely due to the fact that hydrogen peroxide could be deactivated by reacting with acid in the gastric lumen or that metals such as iron and copper might be sequestrated in gastric mucus [5].

## Conclusion

Colonic pseudolipomatosis is a benign and rare condition of which many physicians are unaware. The diagnosis is usually made after a confrontation of endoscopic and histopathological characteristics. Pathogenesis is controversial and includes intramucosal penetration of air after mechanical, infectious or chemical mucosal injury. The detection of these lesions should incite to a careful check of the disinfection process with especially the rinse cycle of the endoscope channels, thus minimizing patients' exposure to residual disinfecting agents in the endoscope and avoid further unnecessary investigations.
